# Sex and Racial Disparities in Outcomes of Transcatheter Edge-to-Edge Mitral Valve Repair for Functional Mitral Regurgitation: A Multicenter Prospective Analysis

**DOI:** 10.1016/j.shj.2026.100831

**Published:** 2026-03-03

**Authors:** Kyeeun Park, Amanda Stebbins, Sunwoo Han, Pratik Manandhar, Karan Pahuja, Pyi Phyo Aung, Sreekanth Vemulapalli, Houman Khalili

**Affiliations:** a Division of Cardiology, Memorial Healthcare System, Hollywood, Florida, USA; b Duke Clinical Research Institute, Duke University School of Medicine, Durham, North Carolina, USA; c Department of Biostatistics, Robert Stempel College of Public Health and Social Work, Florida International University, Miami, Florida, USA; d Division of Cardiology, Florida Atlantic University, Boca Raton, Florida, USA

**Keywords:** Functional mitral regurgitation (FMR), Heart failure (HF), Mitral transcatheter edge-to-edge repair (M-TEER), Racial disparities

## Abstract

**Background:**

Functional mitral regurgitation (FMR) is associated with poor heart failure (HF) outcomes. Mitral transcatheter edge-to-edge repair (M-TEER) is recommended for symptomatic patients despite medical therapy, though the influence of sex and race on outcomes remains understudied.

**Methods:**

We analyzed Society of Thoracic Surgeons/American College of Cardiology Transcatheter Valve Therapeutics registry data on patients undergoing M-TEER for FMR (November 2013 to December 2021). Primary outcomes were 30-day and 12-month all-cause and HF hospitalizations and mortality. Cox models adjusted for demographic and clinical variables.

**Results:**

A total of 9441 patients were enrolled (57.4% male; 80.4% White, 16.3% Black, 2.7% Asian). Black patients were younger, had lower left ventricular (LV) ejection fraction, and had larger LV dimensions compared to White patients. They experienced lower 30-day procedural success rates (48.9% vs. 57.0%; *p* < 0.001) and higher adjusted 12-month HF hospitalizations (adjusted hazard ratio 1.35; 95% CI 1.09-1.67; *p* = 0.006). Asian patients generally demonstrated outcomes comparable to White patients. Female patients had higher baseline LV ejection fraction and smaller ventricular dimensions but demonstrated lower 30-day procedural success (48.1% vs. 61.3%; *p* < 0.001) and higher unadjusted 30-day readmission rates. However, adjusted 1-year outcomes were like males.

**Conclusions:**

Racial and sex disparities in M-TEER outcomes are evident. Black patients present with more advanced disease and experience lower procedural success and higher 12-month HF readmission rates compared to White. Female patients demonstrate lower procedural success but similar long-term adjusted outcomes compared to males. Efforts to address these disparities are essential to improving outcomes for all FMR patients.

## Highlights

• Significant sex and racial disparities exist in mitral transcatheter edge-to-edge repair outcomes for functional mitral regurgitation.
• Black patients presented with more advanced cardiomyopathy at baseline.
• Black patients had higher adjusted 1-year heart failure readmission rates.
• Female patients had lower success rates despite better baseline left ventricular parameters.
• Lower procedural success in women was driven by higher residual gradients.

## Graphical abstract

**Figure:**
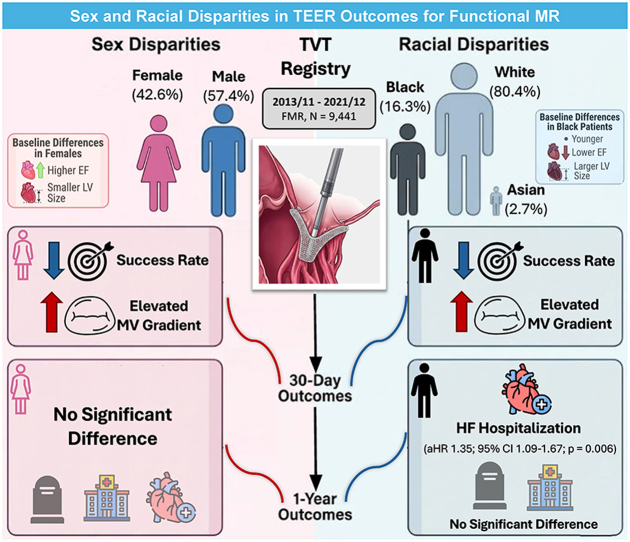

## Introduction

Functional mitral regurgitation (FMR) arises as a consequence of left ventricular (LV) dysfunction and remodeling, leading to apical displacement of the papillary muscles. These pathological changes lead to mitral valve (MV) leaflet tethering, annular dilatation and flattening, and restricted closure of the MV, resulting in mitral regurgitation.^1^ FMR, ranging from moderate to severe, is observed in at least 30% of patients with heart failure (HF) with reduced ejection fraction. It is associated with adverse outcomes, including increased all-cause mortality and HF readmissions.^2^, ^3^, ^4^, ^5^, ^6^ Although guideline-directed medical therapy (GDMT) remains the cornerstone of management, it is often insufficient to fully eliminate regurgitation, particularly in cases of severe refractory disease.^7^

For patients with a low ejection fraction (<50%) who remain symptomatic despite optimized GDMT, mitral transcatheter edge-to-edge valve repair (M-TEER) may provide clinical benefit. The 2022 American Heart Association/American College of Cardiology (ACC)/Heart Failure Society of America guidelines for HF endorse M-TEER as a class 2B recommendation.^8^ This recommendation primarily stems from the Cardiovascular Outcomes Assessment of the MitraClip Percutaneous Therapy for Heart Failure Patients with Functional Mitral Regurgitation (COAPT) trial, which demonstrated that M-TEER significantly reduced all-cause mortality and HF hospitalizations at 24-months follow-up alongside subsequent 5-year data showing persistent and robust clinical benefit.^9^^,^^10^

Despite these established benefits, data regarding the influence of sex and race on M-TEER outcomes remain limited. Females often exhibit distinct mitral anatomy and pathophysiology that may influence repair durability.^11^^,^^12^ Racial disparities in the utilization and effectiveness of structural heart interventions are well documented.^13^^,^^14^ This study aims to investigate the specific impact of sex and race on clinical outcomes following M-TEER for FMR with the goal of refining patient selection and management strategies.

## Methods

### Data Source

The study population was derived from the Society of Thoracic Surgeons/ACC Transcatheter Valve Therapeutics (TVT) Registry, which collects data on all transcatheter MV therapies performed in the United States outside of clinical trials. The research was conducted on deidentified data from the TVT registry. The TVT Registry Protocol was reviewed by Advarra, the ACC Foundation’s Institutional Review Board (IRB) of record. The TVT Registry Protocol was approved as nonexempt human subject research by the IRB. In accordance with 45 Code of Federal Regulations 46.116(d) of the federal regulations, the IRB waived the requirement for obtaining consent. The IRB also waived Health Insurance Portability and Accountability Act (HIPAA) Authorization in accordance with 45 CFR 164.512(i) (2).

### Study Cohort

The study cohort for this analysis included patients who underwent M-TEER with solely functional MV etiology between November 2013 and December 31, 2021. Demographic data on sex and race were self-reported by patients. Patients with unreported sex were excluded from the sex analysis. Patients with unreported races or multiple reported races were excluded from the race analysis.

### Outcomes

The primary outcomes were defined as hospitalization for any cause, hospitalization for HF, and death at 30 and 365 days. HF hospitalization was counted based on the number of patients experiencing at least one admission for HF. Successful repair at 30 days was defined based on Mitral Valve Academic Research Consortium device success criteria: successful device deployment, no conversion to open-heart surgery, survival of the procedure (no intraprocedural or in-laboratory mortality), a 30-day relative mitral regurgitation reduction of ≥1 grade, a mean MV gradient <5 mmHg, and an absolute MR grade of moderate or less at 30 days.

### Statistical Analysis

Categorical variables were summarized as counts and percentages, whereas continuous variables were summarized as means with SDs or medians with 25th and 75th percentiles. The reported status for 1-year mortality, all-cause readmission, and HF readmission ranged from 68% to 89% of eligible procedures (Supplemental Tables 1 and 2). The inverse probability weighting method was used to handle missing data for these outcomes by using weights derived from predicted probabilities from a multivariable logistic model. These weights were included in Cox proportional hazard models that were adjusted for age, gender, race, ethnicity, height, weight, hypertension, diabetes, coronary artery disease, prior stroke, any chronic lung disease, dialysis, glomerular filtration rate, recent HF, LV ejection fraction (LVEF), New York Heart Association class, atrial fibrillation/flutter, prior pacemaker, peripheral artery disease, smoking, home oxygen use, prior myocardial infarction, endocarditis, prior implantable cardioverter defibrillator (ICD), percutaneous coronary intervention, coronary artery bypass grafting, other prior surgeries, acuity status, and hemoglobin levels. With tests for linearity of continuous variables and proportional hazards assumptions, both unadjusted and adjusted Cox proportional hazards models were used to evaluate outcomes. As a sensitivity analysis, multiple imputation was performed to address missing endpoints for death, readmission, and HF readmission. Ten imputed data sets were analyzed separately, and estimates were pooled into a single set of statistics. Spline transformations were applied as needed, and robust SEs were used to account for hospital-level clustering. Adjustments were made based on the predefined covariates as previously described. For nonfatal events, including all-cause and HF readmissions, the Fine–Gray subdistribution hazard model was used to account for the competing risk of death. Cumulative incidence rates at 1 year were calculated using the Kalbfleisch–Prentice cumulative incidence function estimator. Although accounting for slight discrepancies in the total number of cases across 1-year analyses, we found that the date of last contact for nonfatal endpoints differed from the date of last known vital status. Hazard ratios (HRs) with 95% CIs were reported. All statistical analyses were performed using SAS software (version 9.4; SAS Institute, Inc, Cary, North Carolina).

## Results

### Patient Characteristics at Baseline

**Figure 1:**
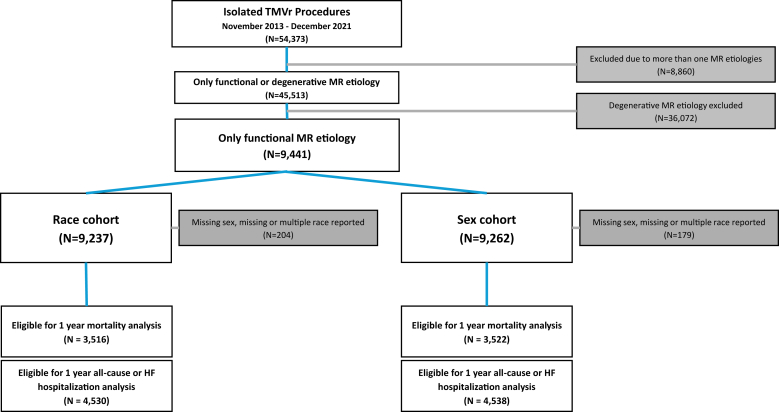
Study flow chart. Abbreviations: HF, heart failure; MR, mitral regurgitation; TMVr, transcatheter mitral valve repair.

From November 2013 through December 31, 2021, a total of 45,513 patients were screened, and 9441 patients were indexed for isolated FMR (Figure 1). In the sex cohort, 5321 patients (57.4%) were male, and 3941 (42.6%) were female. In the race cohort, the majority of patients were identified as White (7424; 80.4%), followed by Black (1504; 16.3%), Asian (246; 2.7%), and American Indian (41; 0.4%).

Tables 1 and 2 outline the demographic and clinical characteristics of the patient cohort. Mitral regurgitation severity was moderate to severe in 19.3% of patients and severe in 74.3%. A substantial portion of patients were on GDMT; 79.8% of patients were receiving beta-blockers, 44.7% were receiving angiotensin-converting enzyme inhibitors/angiotensin II receptor blockers, 14.7% were receiving angiotensin receptor-neprilysin inhibitors, and 24.1% were receiving aldosterone antagonists. Notably, 5125 patients (62.5%) had experienced at least one HF-related hospitalization within the prior year.

**Table 1:** Baseline characteristics by race

Characteristic	All patients (N = 9237)	White (N = 7424)	Black (N = 1504)	*p* value (Black vs. White)	Asian (N = 246)	*p* value (Asian vs. White)	Others (N = 63)
Age, y	72.8 ± 11.4	74.4 ± 10.5	65.3 ± 12.6	<0.001	72.0 ± 11.4	<0.001	67.6 ± 11.8
Male	5305 (57.4)	4409 (59.4)	717 (47.7)	<0.001	143 (58.1)	0.740	36 (57.1)
History							
Hypertension	7935 (85.9)	6323 (85.2)	1364 (90.7)	<0.001	197 (80.4)	0.350	51 (81.0)
Diabetes	3384 (36.7)	2613 (35.2)	625 (41.6)	<0.001	112 (45.7)	0.001	34 (54.0)
Atrial fibrillation/flutter	5528 (59.9)	4740 (63.9)	623 (41.5)	<0.001	130 (52.8)	<0.001	35 (55.6)
GFR, mL/min/1.73 m^2^	51.8 ± 24.6	51.3 ± 23.3	55.1 ± 29.9	<0.001	47.0 ± 26.7	0.005	51.8 ± 27.7
Prior MI	3341 (36.2)	2790 (37.6)	428 (28.5)	<0.001	96 (39.0)	0.690	27 (43.5)
Prior CABG	2542 (27.5)	2267 (30.5)	187 (12.5)	<0.001	75 (30.6)	1.000	13 (20.6)
Prior PCI	3518 (38.1)	3004 (40.5)	394 (26.2)	<0.001	98 (40.0)	0.896	22 (34.9)
ICD	3298 (35.7)	2549 (34.3)	660 (43.9)	<0.001	64 (26.1)	0.008	25 (39.7)
HF in 2 wk	7634 (85.8)	6101 (85.4)	1268 (87.0)	0.052	214 (89.9)	0.062	51 (85.0)
HF hospitalization in 1 y	5125 (62.5)	4017 (61.3)	939 (68.8)	<0.001	141 (63.2)	0.350	28 (48.3)
NYHA class in 2 wk				0.135		0.008	
I	118 (1.3)	99 (1.3)	12 (0.8)		5 (2.0)		2 (3.2)
II	1320 (14.4)	1069 (14.5)	195 (13.0)		48 (19.6)		8 (12.9)
III	5592 (61.0)	4506 (61.2)	929 (62.1)		124 (50.6)		33 (53.2)
IV	2138 (23.3)	1692 (23.0)	359 (24.0)		68 (27.8)		19 (30.6)
Home medications							
Beta blockers	7319 (79.8)	5823 (79.0)	1252 (83.6)	<0.001	192 (79.0)	1.000	52 (82.5)
ACEi/ARB	4094 (44.7)	3279 (44.5)	670 (44.8)	0.059	110 (45.3)	0.784	35 (55.6)
ARNI inhibitor	1001 (14.7)	722 (13.3)	245 (20.6)	<0.001	26 (15.9)	0.410	8 (17.8)
Aldosterone antagonist	2208 (24.1)	1675 (22.7)	467 (31.2)	<0.001	55 (22.6)	1.000	11 (17.5)
Anticoagulant	4405 (48.0)	3738 (50.7)	537 (35.9)	<0.001	102 (42.0)	0.009	28 (44.4)
Aspirin	5000 (54.5)	4025 (54.6)	822 (54.9)	0.780	123 (50.6)	0.240	30 (47.6)
Echocardiogram findings							
LVEF (%)	36.8 ± 15.0	37.9 ± 15.1	31.3 ± 13.5	<0.001	36.2 ± 14.5	0.078	31.9 ± 12.3
LVESV (mL)	113.0 ± 66.0	108.3 ± 64.0	137.3 ± 70.9	<0.001	101.2 ± 55.7	0.177	130.7 ± 82.8
LVEDV (mL)	169.4 ± 77.6	164.3 ± 75.9	195.5 ± 80.9	<0.001	155.8 ± 67.0	0.179	195.6 ± 99.1
LVIDD (cm)	5.8 ± 1.0	5.8 ± 1.0	6.1 ± 1.0	<0.001	5.7 ± 1.1	0.322	6.1 ± 1.2
Mitral regurgitation				<0.001		0.300	
Mild	36 (0.4)	31 (0.4)	4 (0.3)		1 (0.4)		0 (0.0)
Moderate	540 (5.9)	458 (6.2)	68 (4.6)		8 (3.3)		6 (9.5)
Moderate/severe	1767 (19.3)	1460 (19.8)	242 (16.2)		50 (20.3)		15 (23.8)
Severe	6817 (74.3)	5412 (73.4)	1176 (78.8)		187 (76.0)		42 (66.7)

*Notes.* Values are mean ± SD or n (%).

Percentages for baseline clinical characteristics are based on non-missing data for the specific variable.

Abbreviations: ACEi, angiotensin-converting enzyme inhibitor; ARB, angiotensin II receptor blocker; ARNI, angiotensin receptor-neprilysin inhibitor; CABG, coronary artery bypass grafting; GFR, glomerular filtration rate; HF, heart failure; ICD, implantable cardioverter defibrillator; LVEDV, left ventricular end diastolic volume; LVEF, left ventricular ejection fraction; LVESV, left ventricular end systolic volume; LVIDD, left ventricular internal diastolic dimension; MI, myocardial infarction; NYHA, New York Heart Association; PCI, percutaneous coronary intervention.

**Table 2:** Baseline characteristics by sex

Characteristic	All patients (N = 9262)	Male (N = 5321)	Female (N = 3941)	*p* value
Age, y	72.9 ± 11.4	72.7 ± 11.2	73.1 ± 11.7	0.145
Race				<0.001
White	7424 (80.4)	4409 (83.1)	3015 (76.7)	
Black	1504 (16.3)	717 (13.5)	787 (20.0)	
Asian	246 (2.7)	143 (2.7)	103 (2.6)	
Others	63 (0.6)	36 (0.7)	27 (0.7)	
History				
Hypertension	7955 (85.9)	4590 (86.3)	3365 (85.4)	0.222
Diabetes	3395 (36.7)	1988 (37.4)	1407 (35.7)	0.099
Atrial fibrillation/flutter	5544 (59.9)	3357 (63.2)	2187 (55.5)	<0.001
GFR, mL/min/1.73 m^2^	51.8 ± 24.7	52.9 ± 25.2	50.5 ± 23.8	<0.001
Prior MI	3350 (36.2)	2273 (42.8)	1077 (27.4)	<0.001
Prior CABG	2550 (27.6)	1916 (36.0)	634 (16.1)	<0.001
Prior PCI	3527 (38.1)	2374 (44.6)	1153 (29.3)	<0.001
ICD	3306 (35.7)	2202 (41.4)	1104 (28.0)	<0.001
HF in 2 wk	7651 (85.7)	4423 (86.3)	3228 (85.0)	0.090
HF hospitalization in 1 y	5137 (62.5)	2962 (62.7)	2175 (62.3)	0.679
NYHA class in 2 wk				0.074
I	118 (1.3)	61 (1.2)	57 (1.5)	
II	1323 (14.4)	785 (14.9)	538 (13.7)	
III	5606 (61.0)	3171 (60.1)	2435 (62.2)	
IV	2146 (23.3)	1262 (23.9)	884 (22.6)	
Home medications				
Beta blockers	7338 (79.8)	4205 (79.6)	3133 (80.0)	0.641
ACEi/ARB	4108 (44.7)	2361 (44.7)	1747 (44.6)	0.902
ARNI inhibitor	1003 (14.7)	611 (15.5)	392 (13.5)	0.021
Aldosterone antagonist	2216 (24.1)	1246 (23.6)	970 (24.8)	0.193
Anticoagulant	4414 (48.0)	2631 (49.8)	1783 (45.5)	<0.001
Aspirin	5014 (54.5)	3076 (58.2)	1938 (49.5)	<0.001
Echocardiogram findings				
LVEF (%)	36.8 ± 15.0	34.5 ± 13.9	39.8 ± 15.9	<0.001
LVESV (mL)	113.0 ± 66.1	128.2 ± 66.8	91.5 ± 58.7	<0.001
LVEDV (mL)	169.4 ± 77.6	188.9 ± 77.4	142.1 ± 69.2	<0.001
LVIDD (cm)	5.8 ± 1.0	6.1 ± 1.0	5.5 ± 1.0	<0.001
Mitral regurgitation				0.559
Mild	37 (0.4)	20 (0.4)	17 (0.4)	
Moderate	541 (5.9)	300 (5.7)	241 (6.2)	
Moderate/severe	1775 (19.3)	1017 (19.2)	758 (19.4)	
Severe	6832 (74.2)	3936 (74.5)	2896 (74.0)	

*Notes.* Values are mean ± SD or n (%).

Percentages for baseline clinical characteristics are based on non-missing data for the specific variable.

Abbreviations: ACEi, angiotensin-converting enzyme inhibitor; ARB, angiotensin II receptor blocker; ARNI, angiotensin receptor-neprilysin inhibitor; CABG, coronary artery bypass grafting; GFR, glomerular filtration rate; HF, heart failure; ICD, implantable cardioverter defibrillator; LVEDV, left ventricular end diastolic volume; LVEF, left ventricular ejection fraction; LVESV, left ventricular end systolic volume; LVIDD, left ventricular internal diastolic dimension; MI, myocardial infarction; NYHA, New York Heart Association; PCI, percutaneous coronary intervention.

Racial comparisons revealed distinct differences. Black patients were generally younger and included a higher proportion of females than White patients. They presented with lower LVEF (31.3% ± 13.5% vs. 37.9% ± 15.1% in White patients; *p* < 0.001) and larger LV size (LV end-diastolic volumes: 195.5 ± 80.9 mL vs. 164.3 ± 75.9 mL in White patients; *p* < 0.001). In addition, Black patients had a significantly higher rate of HF hospitalization in the preceding year compared to White patients (68.8% vs. 61.3%; *p* < 0.001). This was accompanied by higher utilization rates of device therapy and GDMT, specifically regarding ICDs (43.9% vs. 34.3%; *p* < 0.001), beta-blockers (83.6% vs. 79.0%; *p* < 0.001), angiotensin receptor-neprilysin inhibitors (20.6% vs. 13.3%; *p* < 0.001), and aldosterone antagonists (31.2% vs. 22.7%; *p* < 0.001). There was similar usage of angiotensin-converting enzyme inhibitors/angiotensin II receptor blockers (44.8% vs. 44.5%; *p* = 0.059).

Regarding sex differences, female patients showed higher LVEF (39.8% ± 15.9% in females vs. 34.5% ± 13.9% in males; *p* < 0.001) but smaller LV sizes (LV end-diastolic volume: 142.1 ± 69.2 mL vs. 188.9 ± 77.4 mL in males; *p* < 0.001).

### Procedural Data

The detailed procedural and 30-day outcomes are presented in Supplemental Tables 3 and 4. In-hospital complications, including bleeding, strokes, and conversion to cardiac surgery, were rare (<1.0%). The overall 30-day success rate was lower compared to other studies due to the inclusion of a MV gradient threshold below 5 mmHg as a criterion. Specifically, the success rate was 87.4% when the MV gradient was not included and 55.7% when the MV gradient was factored into the analysis. Following the COAPT trial, the annualized volume of M-TEER procedures increased substantially from 268 cases per year in the pre-COAPT era (January 2013-August 2018) to 2324 cases per year in the post-COAPT era (September 2018-December 2021), representing an 8.7-fold increase. Although there were no significant differences in sex distribution between the 2 eras (*p* = 0.690), the representation of Black patients within the total cohort increased significantly from 12.2% in the pre-COAPT era to 17.1% in the post-COAPT era (*p* < 0.001) (Supplemental Table 5).

### Outcomes by Race

The 30-day procedural outcomes, presented in Table 3, demonstrated a significantly lower success rate among Black patients (48.9%) compared to White patients (57.0%; *p* < 0.001) and Asian patients (57.7%). This disparity was primarily driven by a higher incidence of elevated MV gradients in Black patients (42.3%) compared to White patients (35.6%; *p* < 0.001). However, even when the gradient criterion was excluded, the success rate remained statistically lower in Black patients (84.4% vs. 87.9%; *p* = 0.001). Overall, the 30-day mortality rate was 4.1%, the readmission rate was 9.3%, and HF readmission was 4.5%, with no significant differences observed between racial groups. One-year outcomes, summarized in Table 4, revealed HF readmission rates of 16.7% for White patients, 24.5% for Black patients, and 13.5% for Asian patients. After adjustment, Black patients had a higher risk of HF readmission compared to White patients (adjusted HR [aHR] 1.35; 95% CI: 1.09-1.67; *p* = 0.006) (Table 5). Sensitivity analyses using multiple imputations yielded results consistent with the primary inverse probability weighting analysis. To evaluate whether the etiology of FMR influenced outcomes, we performed interaction analyses stratified by ischemic versus nonischemic status (Supplemental Table 6). The lack of significant interaction between race and FMR etiology (*p* = 0.631) indicated that the disparity in HF readmission risk for Black patients was consistent across both ischemic and nonischemic subgroups.

**Table 3:** Thirty-day outcome by race

Characteristic	All patients (N = 9237)	White (N = 7424)	Black (N = 1504)	*p* value (Black vs. White)	Asian (N = 246)	*p* value (Asian vs. White)	Others (N = 63)
Procedural success	5145 (55.7)	4233 (57.0)	736 (48.9)	<0.001	142 (57.7)	0.826	34 (54.0)
Elevated MV gradient (>5 mmHg)	3339 (36.8)	2595 (35.6)	622 (42.3)	<0.001	90 (37.3)	0.592	27 (43.5)
Procedure success without MV gradient	8073 (87.4)	6523 (87.9)	1269 (84.4)	0.001	224 (91.1)	0.157	57 (90.5)
Mortality	305 (4.1)	251 (4.2)	40 (3.3)	0.144	11 (5.3)	0.406	3 (5.8)
Readmission	682 (9.3)	559 (9.6)	115 (9.5)	0.972	7 (3.6)	0.006	1 (2.0)
Heart failure readmission	325 (4.5)	258 (4.4)	63 (5.2)	0.223	4 (2.1)	0.118	0 (0.0)

*Notes.* Values are mean ± SD or n (%).

Abbreviation: MV, mitral valve.

**Table 4:** One-year outcomes by race

	All patients	White	Black	*p* value (Black vs. White)	Asian	*p* value (Asian vs. White)	Others
All-cause mortality	873 (24.8)	724 (25.2)	116 (21.5)	0.047	26 (27.3)	0.548	7 (24.0)
Readmission	1327 (36.9)	1043 (35.8)	241 (42.1)	0.008	37 (42.5)	0.542	6 (26.1)
Heart failure readmission	640 (18.6)	488 (16.7)	140 (24.5)	<0.001	12 (13.5)	0.324	0 (0.0)

*Notes.* Values are mean ± SD or n (%).

**Table 5:** Multivariable-adjusted Cox proportional hazard models

	Adjusted hazard ratio 95% CI	*p* Value	Adjusted hazard ratio 95% CI∗	*p* value∗
Mortality				
Black (vs. White)	0.92 (0.73-1.15)	0.442	0.98 (0.80-1.20)	0.831
Asian (vs. White)	1.04 (0.72-1.51)	0.828	1.11 (0.75-1.63)	0.604
Female	0.92 (0.77-1.09)	0.327	0.88 (0.74-1.05)	0.158
Readmission				
Black (vs. White)	1.07 (0.92-1.25)	0.393	1.07 (0.92-1.25)	0.357
Asian (vs. White)	1.05 (0.77-1.43)	0.768	1.02 (0.76-1.38)	0.892
Female	0.99 (0.85-1.14)	0.864	1.01 (0.88-1.17)	0.878
Heart failure readmission				
Black (vs. White)	1.35 (1.09-1.67)	0.006	1.31 (1.07-1.61)	0.011
Asian (vs. White)	0.71 (0.41-1.23)	0.222	0.79 (0.47-1.34)	0.386
Female	1.00 (0.81-1.25)	0.981	1.09 (0.88-1.35)	0.421

∗ Calculated using multiple imputation techniques.

### Outcomes by Sex

Table 6 presents the 30-day outcomes stratified by sex. Female patients experienced a lower success rate compared to male patients (48.1% vs. 61.3%; *p* < 0.001). This disparity was predominantly driven by a higher incidence of elevated MV gradients (45.0% vs. 30.8%; *p* < 0.001), as the difference in success rates narrowed substantially when the gradient criterion was excluded (86.5% vs. 88.0%; *p* = 0.032). In addition, we observed higher 30-day all-cause readmission rates (10.3% vs. 8.7%; *p* = 0.017) and increased HF readmission rates (5.0% vs. 4.0%; *p* = 0.033) in female patients. However, the overall incidence of these events was low, and the absolute difference between groups was small. One-year outcomes are detailed in Table 7. At 12-month follow-up, HF admission rates were 17.8% in males and 20.0% in females. After adjustment, there was no significant difference (aHR 1.00; 95% CI: 0.81-1.25; *p* = 0.981) (Table 5). These primary outcomes remained consistent after sensitivity and interaction analyses (Supplemental Table 6).

**Table 6:** Thirty-day outcome by sex

Characteristic	All patients (N = 9262)	Male (N = 5321)	Female (N = 3941)	*p* value
Procedural success	5155 (55.7)	3260 (61.3)	1895 (48.1)	<0.001
Elevated MV gradient (>5 mmHg)	3346 (36.8)	1612 (30.8)	1737 (45.0)	<0.001
Procedure success without MV gradient	8095 (87.4)	4685 (88.0)	3410 (86.5)	0.032
Mortality	307 (4.1)	183 (4.2)	124 (3.9)	0.460
Readmission	685 (9.4)	364 (8.7)	321 (10.3)	0.017
Heart failure readmission	325 (4.4)	168 (4.0)	157 (5.0)	0.033

*Notes.* Values are mean ± SD or n (%).

Abbreviation: MV, mitral valve.

**Table 7:** One-year outcomes by sex

	All patients	Male	Female	*p* value
All-cause mortality	877 (24.7)	532 (26.1)	345 (23.0)	0.020
Readmission	1343 (38.4)	752 (37.4)	591 (39.7)	0.152
Heart failure readmission	647 (18.8)	355 (17.8)	292 (20.0)	0.117

*Notes.* Values are mean ± SD or n (%).

## Discussion

Focusing on differences by sex and race, this study evaluated the characteristics and 12-month outcomes of patients undergoing M-TEER for isolated FMR. Among racial groups, Black patients exhibited distinct clinical characteristics—including younger age, a higher proportion of females, lower LVEF, and larger LV size—compared to White patients. The 30-day procedural success rate was 8.1% lower in Black patients, who also had a higher proportion of elevated MV gradient by 6.7% compared to White patients. At 12 months, Black patients had an increased adjusted risk of HF readmission (aHR vs. White 1.35; 95% CI: 1.09-1.67; *p* = 0.006). Female patients had higher LVEF and smaller LV size at baseline. Nevertheless, the 30-day procedural success rate was 13.2% lower in females, who also had a 14.2% higher proportion of elevated MV gradient. In addition, females experienced higher rates of all-cause and HF readmissions within 30 days, although the findings were not adjusted for confounding factors and a small overall incidence difference; this makes the clinical implication uncertain.

To the best of our knowledge, this is the first multicenter prospective study to investigate both index and follow-up outcomes by sex and race in patients undergoing M-TEER for FMR. Previous studies have often relied on administrative and diagnosis code–based data or single-center analyses. In contrast, we collected prospective, multicenter, physician-curated data that better reflect real-world practices regarding M-TEER. The comprehensive information on patient characteristics, including GDMT, echocardiographic parameters, and comorbidities (such as concomitant kidney and lung diseases), enabled us to minimize confounding factors other than race and sex.

As previously reported, our study demonstrated racial disparities in M-TEER. Black patients constituted a larger proportion (16.3%) of our cohort—aligning more closely with the national demographic distribution (13.8% in 2020)—compared to prior studies, which reported 7.4% to 10.0% of study populations being Black.^13^, ^14^, ^15^, ^16^, ^17^ However, the representation of Asian (2.7%) and Hispanic (7.3%) patients was lower than their national demographic proportions (6.2% and 18.7% in 2020, respectively).^17^ This discrepancy may reflect structural inequities in access to minimally invasive procedures as with non-Hispanic Black patients, who are less likely to have private insurance or undergo these procedures even when insured.^15^ As evidenced by their lower LVEF, higher HF admissions within a year, and larger LV size, Black patients were also often referred for M-TEER at more advanced stages of HF than White patients. This disparity may reflect structural racism and implicit bias that hinder optimal care for the Black population.^18^^,^^19^

Our study also revealed that female patients had higher LVEF and lower LV size compared to male patients. This observation aligns with sex-based differences documented in other M-TEER studies, including subgroup analyses of the COAPT trial.^9^^,^^20^, ^21^, ^22^ The echocardiographic assessment of MR severity is based on effective regurgitant orifice area and regurgitant volume. However, because these parameters are not indexed or adjusted for sex, MR severity in women may be underestimated. Given their smaller LV size, an equivalent regurgitant volume could be more detrimental in women than in men.^20^ In addition, the larger LV size in male patients often facilitates the exclusion of severe FMR cases from M-TEER procedures, whereas “end-stage” LV disease in women may not be excluded as frequently due to their generally smaller LV size.^20^ Although pinpointing the exact causes of differences in procedural success rates within this complex patient cohort is challenging, even with higher LVEF in our study, these factors may contribute to the observed lower 30-day success rate, higher rate of elevated MV gradient, and increased 30-day rehospitalization rates seen in women. Future research incorporating sex-specific cutoffs for M-TEER in FMR could provide further insights into these disparities.

A key finding of our analysis is that Black patients experienced significantly higher HF readmission rates at 1 year compared to White patients (aHR 1.35). Importantly, this disparity persisted even after adjustment for demographics and clinical risk factors and, notably, despite the higher baseline utilization of GDMT and device therapies observed in this cohort. This suggests that additional race-related factors not captured in our study may influence the long-term prognosis of M-TEER in FMR. Black patients with HF are documented to be less likely to receive optimal medical treatment, advanced therapies, or devices and are more prone to frequent hospitalizations.^23^ However, Black patients in our study actually demonstrated comparable or higher rates of GDMT and ICD utilization relative to other races. Furthermore, although Black patients had a higher burden of HF hospitalizations in the year before M-TEER, our adjusted analysis indicates that this history alone did not fully account for the increased event rate during follow-up, pointing to the influence of complex variables such as advanced disease status, socioeconomic factors, adherence to treatment, and structural disparities. Notably, Black patients tend to have comparable or better outcomes than White patients when early posthospital follow-up is implemented.^24^^,^^25^ Encouragingly, we observed that the utilization of M-TEER among Black patients significantly increased following the COAPT trial. Our findings underscore the critical importance of early intervention and hospital follow-up, particularly for minority groups such as Black patients.

Our study confirmed that M-TEER for FMR is equally safe and effective across sexes and races in the real world, as evidenced by low in-hospital mortality, readmissions within 30 days, 1-year mortality, and HF hospitalization rates. This is reflected in the significant decrease in overall 1-year HF hospitalization rates before and after M-TEER (62.5% vs. 18.6%-18.8%). In addition, similar to our findings, single-center retrospective studies reported a 15% overall HF hospitalization rate after M-TEER,^13^ which is substantially lower than the rates observed in the Multicentre Randomized Study of Percutaneous Mitral Valve Repair Cardiovascular Outcomes Using the MitraClip System in Patients with Severe Secondary Mitral Regurgitation (MITRA-FR) study (48.7% in the intervention group and 47.4% in the control group).^26^ One-year mortality after M-TEER in previous studies ranged from 17.9% to 25%,^12^^,^^13^^,^^20^^,^^21^ which is consistent with our study (overall 1-year mortality was 21.5% in Black patients, 25.2% in White patients, and 27.3% in Asian patients). As observed in the 5-year follow-up of the COAPT trial, M-TEER is believed to provide durable mortality benefits in the long term.^10^

Our study has several limitations. First, by only including patients who identified as a single race, we failed to account for the possibility of mixed racial backgrounds. Although unlikely, there could be errors in self-reported race data. Second, there were high rates of missing data at the 1-year follow-up; in particular, 32% of mortality data and 12% of admission data were missing. However, the similar distribution of these dropouts among the races and sexes make survival bias less likely (Supplemental Tables 3 and 4), and the primary outcomes remained consistent after application of multiple imputation techniques for missing data. Third, the lack of availability of data regarding postprocedural adherence to GDMT may have influenced long-term outcomes. In addition, given that the study period largely predated the universal adoption of sodium-glucose cotransporter-2 inhibitors as guideline-directed therapy for HF, data on their utilization were not captured. Fourth, the lack of availability of socioeconomic data such as insurance status, income, or area of residence in our study limits our ability to fully account for disparities in medical access. Fifth, due to small sample sizes, robust multivariable Cox proportional hazards analyses comparing other racial groups (including Asian and Hispanic patients) to White patients could not be performed. Finally, this is a prospective observational analysis that is inherently limited by residual confounding and selection bias. Future studies are needed to evaluate disparities in M-TEER outcomes across sex and race.

## Conclusion

In this multicenter prospective analysis, racial and sex disparities were evident in the application and outcomes of M-TEER for FMR. Most notably, Black patients faced a significantly higher risk of 1-year HF readmission compared to White patients, a disparity that remained significant after adjustment for confounding factors. In addition, Black patients presented with more advanced disease stages, which likely contributed to their lower 30-day procedural success rates. Female patients—characterized by smaller LV size and higher LVEF—also exhibited lower procedural success rates, likely due to anatomical constraints. Despite these disparities, as evidenced by low in-hospital mortality, 30-day HF readmission, 1-year mortality, and 1-year HF hospitalization, our study confirmed that M-TEER is a safe and effective treatment for FMR across different sexes and races in real-world clinical practice. These findings underscore the need for tailored, patient-specific management strategies and highlight the importance of addressing structural inequities in health care to improve outcomes for all patients with FMR.

## Ethics Statement

The research reported in this manuscript adhered to all relevant ethical guidelines, including the Declaration of Helsinki. The TVT Registry Protocol was reviewed by Advarra, the American College of Cardiology Foundation's Institutional Review Board (IRB) of record, and was approved as nonexempt human subject research. In accordance with 45 Code of Federal Regulations (CFR) 46.116(d), the IRB waived the requirement for obtaining informed consent. The IRB also waived Health Insurance Portability and Accountability Act (HIPAA) Authorization in accordance with 45 CFR 164.512(i)(2).

## Funding

The authors have no funding to report.

## Disclosure Statement

Houman Khalili has received research grants and speaker fees from Edwards Life Sciences. Sreekanth Vemulapalli has received grants/contracts from the American College of Cardiology, Society of Thoracic Surgeons, National Institutes of Health, Cytokinetics, Abbott Vascular, and the Food and Drug Administration; and consulting/honoraria from Medtronic, Veralox Therapeutics, American College of Physicians, AstraZeneca, Boehringer Ingelheim, Cytokinetics, and Icon.

The other authors had no conflicts to declare.
