# Supplementary material for: Sex and Racial Disparities in Outcomes of Transcatheter Edge-to-Edge Mitral Valve Repair for Functional Mitral Regurgitation: A Multicenter Prospective Analysis
Source: Struct Heart. 2026 Mar 3;10(5):100831. doi: 10.1016/j.shj.2026.100831 (PMC13112474; doi:10.1016/j.shj.2026.100831)
Supplement: Supplemental Tables 1-6 [file mmc1.docx]

# **Supplemental Table 1(a). Baseline Characteristics of the Race Analysis Cohort Stratified by One-Year Vital Status Reporting Status**

| **Characteristic** | **All Patients (N = 5,163)** | **Reported  (N = 3,516)** | **Not Reported  (N = 1,647)** |
| --- | --- | --- | --- |
| Age, years | 72.7 ± 11.7 | 72.5 ± 11.8 | 73.1 ± 11.6 |
| Male | 2,992 (58.0) | 2,011 (57.2) | 981 (59.6) |
| Race | | | |
| White | 4,182 (81.0) | 2,858 (81.3) | 1,324 (80.4) |
| Black | 801 (15.5) | 540 (15.4) | 261 (15.8) |
| Asian | 144 (2.8) | 93 (2.6) | 51 (3.1) |
| Other | 36 (0.7) | 25 (0.7) | 11 (0.7) |
| **History** | | | |
| Hypertension | 4,402 (85.3) | 3,004 (85.5) | 1,398 (84.9) |
| Diabetes | 1,902 (36.9) | 1,299 (37.0) | 603 (36.6) |
| Atrial fibrillation/flutter | 2,981 (57.8) | 2,079 (59.2) | 902 (54.9) |
| GFR | 51.1 ± 24.6 | 50.9 ± 24.6 | 51.5 ± 24.7 |
| Prior MI | 1,941 (37.7) | 1,351 (38.5) | 590 (35.9) |
| Prior CABG | 1,490 (28.9) | 1,015 (28.9) | 475 (28.9) |
| Prior PCI | 1,998 (38.7) | 1,384 (39.4) | 614 (37.3) |
| ICD | 1,906 (36.9) | 1,378 (39.2) | 528 (32.1) |
| HF in 2 weeks | 4,493 (87.1) | 3,053 (86.9) | 1,440 (87.5) |
| HF hospitalization in 1 year | 3,092 (66.1) | 2,122 (66.3) | 970 (65.8) |
| NYHA class in 2 weeks |  |  |  |
| I | 58 (1.1) | 39 (1.1) | 19 (1.2) |
| II | 659 (12.9) | 469 (13.5) | 190 (11.6) |
| III | 3,050 (59.5) | 2,076 (59.6) | 974 (59.5) |
| IV | 1,355 (26.5) | 902 (25.9) | 453 (27.7) |
| LVEF (%) | 36.6 ± 15.2 | 36.1 ± 14.8 | 37.6 ± 15.8 |
| Values are mean ± SD or n (%)  CABG: coronary artery bypass grafting; GFR: glomerular filtration rate; HF: heart failure; ICD: implantable cardioverter defibrillator; LVEF: left ventricular ejection fraction; MI: myocardial infarction; NYHA: New York Heart Association; PCI: percutaneous coronary intervention | | | |

**Supplemental Table 1(b). Baseline Characteristics of the Race Analysis Cohort Stratified by One-Year All-Cause and Heart Failure Readmission Reporting Status.**

| **Characteristic** | **All Patients (N = 5,163)** | **Reported (N = 4,530)** | **Not Reported  (N = 633)** |
| --- | --- | --- | --- |
| Age, years | 72.7 ± 11.7 | 72.6 ± 11.8 | 73.3 ± 11.4 |
| Male | 2,992 (58.0) | 2,630 (58.1) | 362 (57.2) |
| Race | | | |
| White | 4,182 (81.0) | 3,670 (81.0) | 512 (80.9) |
| Black | 801 (15.5) | 713 (15.7) | 88 (13.9) |
| Asian | 144 (2.8) | 115 (2.5) | 29 (4.6) |
| Other | 36 (0.7) | 32 (0.7) | 4 (0.6) |
| **History** |  |  |  |
| Hypertension | 4,402 (85.3) | 3,865 (85.3) | 537 (84.8) |
| Diabetes | 1,902 (36.9) | 1,654 (36.5) | 248 (39.2) |
| Atrial fibrillation/flutter | 2,981 (57.8) | 2,630 (58.2) | 351 (55.5) |
| GFR | 51.1 ± 24.6 | 51.3 ± 24.6 | 49.0 ± 25.0 |
| Prior MI | 1,941 (37.7) | 1,351 (38.5) | 590 (35.9) |
| Prior CABG | 1,490 (28.9) | 1,015 (28.9) | 475 (28.9) |
| Prior PCI | 1,998 (38.7) | 1,384 (39.4) | 614 (37.3) |
| ICD | 1,906 (36.9) | 1,378 (39.2) | 528 (32.1) |
| HF in 2 weeks | 4,493 (87.1) | 3,053 (86.9) | 1,440 (87.5) |
| HF hospitalization in 1 year | 3,092 (66.1) | 2,677 (65.2) | 415 (72.8) |
| NYHA class in 2 weeks |  |  |  |
| I | 58 (1.1) | 51 (1.1) | 7 (1.1) |
| II | 659 (12.9) | 600 (13.4) | 59 (9.4) |
| III | 3,050 (59.5) | 2,742 (61.0) | 308 (49.0) |
| IV | 1,355 (26.5) | 1,101 (24.5) | 254 (40.4) |
| LVEF (%) | 36.6 ± 15.2 | 36.5 ± 15.0 | 37.2 ± 16.4 |
| Values are mean ± SD or n (%)  Abbreviations as in ***Supplemental*** [***Table***](https://www.jacc.org/doi/10.1016/j.jcin.2024.08.004#tbl1) ***1(a).*** | | | |

# **Supplemental Table 2(a). Baseline Characteristics of the Sex Analysis Cohort Stratified by One-Year Vital Status Reporting Status.**

| **Characteristic** | **All Patients (N = 5,173)** | **Reported (N = 3,522)** | **Not Reported  (N = 1,651)** |
| --- | --- | --- | --- |
| Age, years | 72.7 ± 11.7 | 72.5 ± 11.8 | 73.1 ± 11.6 |
| Male | 2,998 (58.0) | 2,015 (57.2) | 983 (59.5) |
| Race |  |  |  |
| White | 4,182 (81.0) | 2,858 (81.3) | 1,324 (80.4) |
| Black | 801 (15.5) | 540 (15.4) | 261 (15.8) |
| Asian | 144 (2.8) | 93 (2.6) | 51 (3.1) |
| Other | 36 (0.7) | 25 (0.7) | 11 (0.7) |
| **History** |  |  |  |
| Hypertension | 4,410 (85.3) | 3,008 (85.4) | 1,402 (84.9) |
| Diabetes | 1,902 (36.8) | 1,654 (37.0) | 248 (36.6) |
| Atrial fibrillation/flutter | 2,987 (57.8) | 2,081 (59.2) | 906 (55.0) |
| GFR | 51.1 ± 24.6 | 50.8 ± 24.6 | 51.5 ± 24.7 |
| Prior MI | 1,942 (37.6) | 1,351 (38.5) | 591 (35.9) |
| Prior CABG | 1,492 (28.9) | 1,016 (28.9) | 476 (28.9) |
| Prior PCI | 2,002 (38.7) | 1,386 (39.4) | 616 (37.3) |
| ICD | 1,911 (37.0) | 1,381 (39.2) | 530 (32.1) |
| HF in 2 weeks | 4,500 (87.1) | 3,056 (86.8) | 1444 (87.6) |
| HF hospitalization in 1 year | 3,098 (66.1) | 2,125 (66.2) | 973 (65.8) |
| NYHA class in 2 weeks |  |  |  |
| I | 58 (1.1) | 39 (1.1) | 19 (1.2) |
| II | 661 (12.9) | 471 (13.5) | 190 (11.6) |
| III | 3,053 (59.5) | 2,077 (59.5) | 976 (59.5) |
| IV | 1,360 (26.5) | 905 (25.9) | 455 (27.7) |
| LVEF (%) | 36.6 ± 15.2 | 36.1 ± 14.9 | 37.6 ± 15.9 |
| Values are mean ± SD or n (%)  Abbreviations as in ***Supplemental*** [***Table***](https://www.jacc.org/doi/10.1016/j.jcin.2024.08.004#tbl1) ***1(a).*** | | | |

# **Supplemental Table 2(b). Baseline Characteristics of the Sex Analysis Cohort Stratified by One-Year Readmission Reporting Status.**

| **Characteristic** | **All Patients (N = 5,173)** | **Reported (N = 3,516)** | **Not Reported  (N = 1,657)** |
| --- | --- | --- | --- |
| Age, years | 72.7 ± 11.7 | 72.4 ± 11.8 | 73.3 ± 11.6 |
| Male | 2,998 (58.0) | 2,019 (57.4) | 979 (59.1) |
| Race |  |  |  |
| White | 4,182 (81.0) | 2,842 (80.9) | 1,340 (81.1) |
| Black | 801 (15.5) | 556 (15.8) | 245 (14.8) |
| Asian | 144 (2.8) | 90 (2.6) | 54 (3.3) |
| Other | 36 (0.7) | 23 (0.7) | 13 (0.8) |
| **History** |  |  |  |
| Hypertension | 4,410 (85.3) | 2,994 (85.2) | 1,416 (85.5) |
| Diabetes | 1,902 (36.8) | 1,380 (39.3) | 522 (37.1) |
| Atrial fibrillation/flutter | 2,987 (57.8) | 2,057 (58.6) | 930 (56.2) |
| GFR | 51.1 ± 24.6 | 51.1 ± 24.6 | 50.9 ± 24.6 |
| Prior MI | 1,942 (37.6) | 1,328 (37.9) | 614 (37.1) |
| Prior CABG | 1,492 (28.9) | 1,002 (28.5) | 490 (29.7) |
| Prior PCI | 2,002 (38.7) | 1,373 (39.1) | 629 (38.0) |
| ICD | 1,911 (37.0) | 1,372 (39.1) | 539 (32.5) |
| HF in 2 weeks | 4,500 (87.1) | 3,040 (86.5) | 1,460 (88.3) |
| HF hospitalization in 1 year | 3,098 (66.1) | 2,109 (65.9) | 989 (66.5) |
| NYHA class in 2 weeks |  |  |  |
| I | 58 (1.1) | 41 (1.2) | 17 (1.0) |
| II | 661 (12.9) | 474 (13.6) | 187 (11.4) |
| III | 3,053 (59.5) | 2,131 (61.1) | 922 (56.0) |
| IV | 1,360 (26.5) | 840 (24.1) | 520 (31.6) |
| LVEF (%) | 36.6 ± 15.2 | 36.0 ± 14.7 | 37.7 ± 16.1 |
| Values are mean ± SD or n (%)  Abbreviations as in ***Supplemental*** [***Table***](https://www.jacc.org/doi/10.1016/j.jcin.2024.08.004#tbl1) ***1(a).*** | | | |

# **Supplemental Table 2(c). Baseline Characteristics of the Race Analysis Cohort Stratified by One-Year Heart Failure Readmission Reporting Status.**

| **Characteristic** | **All Patients (N = 5,173)** | **Reported (N = 3,457)** | **Not Reported  (N = 1,716)** |
| --- | --- | --- | --- |
| Age, years | 72.7 ± 11.7 | 72.4 ± 11.8 | 73.3 ± 11.6 |
| Male | 2,998 (58.0) | 1,989 (57.5) | 1,009 (58.8) |
| Race |  |  |  |
| White | 4,182 (81.0) | 2,795 (81.0) | 1,387 (81.1) |
| Black | 801 (15.5) | 545 (15.8) | 256 (15.0) |
| Asian | 144 (2.8) | 89 (2.6) | 55 (3.2) |
| Other | 36 (0.7) | 23 (0.7) | 13 (0.8) |
| **History** |  |  |  |
| Hypertension | 4,410 (85.3) | 2,943 (85.2) | 1,467 (85.5) |
| Diabetes | 1,902 (36.8) | 1,354 (39.2) | 548 (37.4) |
| Atrial fibrillation/flutter | 2,987 (57.8) | 2,023 (58.6) | 964 (56.2) |
| GFR | 51.1 ± 24.6 | 51.2 ± 24.6 | 50.8 ± 24.7 |
| Prior MI | 1,942 (37.6) | 1,314 (38.1) | 628 (36.7) |
| Prior CABG | 1,492 (28.9) | 990 (28.7) | 502 (29.3) |
| Prior PCI | 2,002 (38.7) | 1,358 (39.3) | 644 (37.6) |
| ICD | 1,911 (37.0) | 1,354 (39.2) | 557 (32.5) |
| HF in 2 weeks | 4,500 (87.1) | 2,991 (86.6) | 1,509 (88.1) |
| HF hospitalization in 1 year | 3,098 (66.1) | 2,082 (66.1) | 1,016 (66.1) |
| NYHA Class in 2 weeks |  |  |  |
| I | 58 (1.1) | 38 (1.1) | 20 (1.2) |
| II | 661 (12.9) | 467 (13.6) | 194 (11.4) |
| III | 3,053 (59.5) | 2,100 (61.2) | 953 (56.0) |
| IV | 1,360 (26.5) | 824 (24.0) | 536 (31.5) |
| LVEF (%) | 36.6 ± 15.2 | 36.0 ± 14.7 | 37.8 ± 16.1 |
| Values are mean ± SD or n (%)  Abbreviations as in ***Supplemental*** [***Table***](https://www.jacc.org/doi/10.1016/j.jcin.2024.08.004#tbl1) ***1(a).*** | | | |

# **Supplemental Table 3(a). Procedural Outcomes by Race.**

| **Characteristic** | **All Patients (N = 9,237)** | **White (N = 7,424)** | **Black (N = 1,504)** | ***p* Value**  **(Black vs White)** | **Asian (N = 246)** | ***p* Value**  **(Asian vs White)** | **Other   (N = 63)** |
| --- | --- | --- | --- | --- | --- | --- | --- |
| **Number of clips implanted successfully** |  |  |  | 0.010 |  | 0.941 |  |
| 1 | 5,322 (59.8) | 4,323 (60.4) | 820 (56.7) |  | 146 (60.6) |  | 33 (55.0) |
| >1 | 3,581 (40.2) | 2,834 (39.6) | 625 (43.3) |  | 95 (39.4) |  | 27 (45.0) |
| **Site of clip implant** |  | | | 0.020 |  | 0.417 |  |
| Other | 344 (3.9) | 292 (4.1) | 41 (2.8) |  | 8 (3.4) |  | 3 (4.9) |
| A2P2 | 8,512 (96.1) | 6,819 (95.9) | 1,405 (97.2) |  | 230 (96.6) |  | 58 (95.1) |
| **Complications** | | | | | | | |
| Cardiac arrest | 126 (1.4) | 101 (1.4) | 18 (1.2) | 0.700 | 6 (2.4) | 0.253 | 1 (1.6) |
| Transseptal complication | 22 (0.2) | 14 (0.2) | 8 (0.5) | 0.030 | 0 (0.0) | 1.000 | 0 (0.0) |
| Access site hematoma | 63 (0.7) | 54 (0.7) | 9 (0.6) | 0.700 | 0 (0.0) | 0.340 | 0 (0.0) |
| Converted to open heart surgery | 29 (0.3) | 21 (0.3) | 7 (0.5) | 0.370 | 1 (0.4) | 1.000 | 0 (0.0) |
| MI | 3 (<0.1) | 3 (<0.1) | 0 (0.0) | 0.993 | 0 (0.0) | 1.000 | 0 (0.0) |
| Any stroke | 57 (0.6) | 41 (0.6) | 14 (0.9) | 0.126 | 1 (0.4) | 1.000 | 1 (1.6) |
| Hemorrhagic stroke | 8 (0.1) | 4 (0.1) | 3 (0.2) | 0.180 | 0 (0.0) | 1.000 | 1 (1.6) |
| Ischemic stroke | 45 (0.5) | 35 (0.5) | 9 (0.6) | 0.660 | 1 (0.4) | 1.000 | 0 (0.0) |
| TIA | 8 (0.1) | 7 (0.1) | 1 (0.1) | 1.000 | 0 (0.0) | 1.000 | 0 (0.0) |
| Device embolism | 5 (0.1) | 4 (0.1) | 1 (0.1) | 1.000 | 0 (0.0) | 1.000 | 0 (0.0) |
| Device thrombosis | 1 (<0.1) | 1 (<0.1) | 0 (0.0) | 1.000 | 0 (0.0) | 1.000 | 0 (0.0) |
| In-hospital death | 185 (2.0) | 146 (2.0) | 25 (1.7) | 0.495 | 11 (4.5) | 0.013 | 3 (4.8) |
| **Discharge Location** |  | | | 0.123 |  | 0.318 |  |
| Home | 8,393 (92.7) | 6,734 (92.5) | 1,384 (93.6) |  | 217 (92.3) |  | 58 (96.7) |
| Extended care/rehab | 327 (3.6) | 275 (3.8) | 37 (2.5) |  | 13 (5.5) |  | 2 (3.3) |
| Other acute care hospital | 68 (0.8) | 52 (0.7) | 14 (0.9) |  | 2 (0.9) |  | 0 (0.0) |
| Skilled nursing facility | 200 (2.2) | 166 (2.3) | 32 (2.2) |  | 2 (0.9) |  | 0 (0.0) |
| Hospice care | 26 (0.3) | 23 (0.3) | 3 (0.2) |  | 0 (0.0) |  | 0 (0.0) |
| **Length of stay (days)** |  | | | 0.002 |  | 0.014 |  |
| Mean +/- SD | 5.0 +/- 9.8 | 4.7 +/- 8.5 | 5.9 +/- 14.3 |  | 6.6 +/- 12.0 |  | 6.0 +/- 7.7 |
| Median (25th, 75th) | 1.0 (1.0, 5.0) | 1.0 (1.0, 4.0) | 2.0 (1.0, 6.5) |  | 2.0 (1.0, 7.0) |  | 2.0 (1.0, 11.0) |
| Values are mean ± SD or n (%)  MI: myocardial infarction, TIA: transient ischemic attack | | | | | | | |

# **Supplemental Table 3(b). Procedural Outcomes by Sex.**

| **Characteristic** | **All Patients (N = 9,262)** | **Male (N = 5,321)** | **Female (N = 3,941)** | ***p* Value** | |
| --- | --- | --- | --- | --- | --- |
| **Number of clips implanted successfully** |  |  |  | <0.001 | |
| 1 | 5,338 (59.8) | 2,731 (52.9) | 2,607 (69.2) |  | |
| >1 | 3,589 (40.2) | 2,429 (47.1) | 1,160 (30.8) |  | |
| **Site of clip implant** |  | | | 0.717 | |
| Other | 346 (3.9) | 203 (4.0) | 143 (3.8) |  | |
| A2P2 | 8,534 (96.1) | 4,923 (96.0) | 3,611 (96.2) |  | |
| **Complications** | | | | |  |
| Cardiac arrest | 127 (1.4) | 78 (1.5) | 49 (1.2) | 0.363 | |
| Transseptal complication | 22 (0.2) | 11 (0.2) | 11 (0.3) | 0.480 | |
| Access site hematoma | 63 (0.7) | 25 (0.5) | 38 (1.0) | 0.004 | |
| Converted to open heart surgery | 29 (0.3) | 17 (0.3) | 12 (0.3) | 0.898 | |
| MI | 3 (<0.1) | 1 (<0.1) | 2 (0.1) | 0.579 | |
| Any stroke | 57 (0.6) | 35 (0.7) | 22 (0.6) | 0.545 | |
| Hemorrhagic stroke | 8 (0.1) | 7 (0.1) | 1 (<0.1) | 0.150 | |
| Ischemic stroke | 45 (0.5) | 27 (0.5) | 18 (0.5) | 0.729 | |
| TIA | 8 (0.1) | 2 (<0.1) | 6 (0.2) | 0.0793 | |
| Device embolism | 5 (0.1) | 3 (0.1) | 2 (0.1) | 1.000 | |
| Device thrombosis | 9,261 (100.0) | 5,320 (100.0) | 3,941 (100.0) | 1.000 | |
| In-hospital death | 186 (2.0) | 112 (2.1) | 74 (1.9) | 0.441 | |
| **Discharge location** |  | | | <0.001 | |
| Home | 8,416 (92.7) | 4,854 (93.2) | 3,562 (92.1) |  | |
| Extended care/rehab | 327 (3.6) | 178 (3.4) | 149 (3.9) |  | |
| Other acute care hospital | 68 (0.7) | 43 (0.8) | 25 (0.6) |  | |
| Skilled nursing facility | 201 (2.2) | 91 (1.7) | 110 (2.8) |  | |
| Hospice care | 26 (0.3) | 16 (0.3) | 10 (0.3) |  | |
| **Length of stay (days)** |  | | | 0.388 | |
| n | 9,262 | 5,321 | 3,941 |  | |
| Mean +/- SD | 5.0 +/- 9.8 | 4.9 +/- 8.9 | 5.1 +/- 10.9 |  | |
| Median (25th, 75th) | 1.0 (1.0, 5.0) | 1.0 (1.0, 5.0) | 1.0 (1.0, 5.0) |  | |
| Values are mean ± SD or n (%)  MI: myocardial infarction, TIA: transient ischemic attack | | | | |  |

# **Supplemental Table 4(a). 30-Day Outcomes by Race.**

| **Characteristic** | **All Patients (N = 9,237)** | **White (N = 7,424)** | **Black  (N = 1,504)** | ***p* Value**  **(Black vs White)** | **Asian (N = 246)** | ***p* Value**  **(Asian vs White)** | **Other   (N = 63)** |
| --- | --- | --- | --- | --- | --- | --- | --- |
| Absolute MR of moderate, mild, trace/trivial or none | 8,444 (92.1) | 6,826 (92.5) | 1,332 (89.4) | <0.001 | 228 (93.4) | 0.764 | 58 (92.1) |
| Elevated MV Gradient (>5 mmHg) | 3,339 (36.8) | 2,595 (35.6) | 622  (42.3) | <0.001 | 90 (37.3) | 0.592 | 27 (43.5) |
| Procedural success (MV gradient independent) | 8,073 (87.4) | 6,523 (87.9) | 1,269 (84.4) | <0.001 | 224 (91.1) | 0.157 | 57 (90.5) |
| Procedural success | 5,145 (55.7) | 4,233 (57.0) | 736 (48.9) | <0.001 | 142 (57.7) | 0.877 | 34 (54.0) |
| Values are mean ± SD or n (%)  MR: mitral regurgitation, MV: mitral valve | | | | | | | |

# **Supplemental Table 4(b). 30-Day Outcomes by Sex.**

| **Characteristic** | **All Patients (N = 9,262)** | **Male (N = 5,321)** | **Female (N = 3,941)** | ***p* Value** |
| --- | --- | --- | --- | --- |
| Absolute MR of moderate, mild, trace/trivial or none | 8,467 (92.1) | 4,877 (92.2) | 3,590 (91.8) | 0.360 |
| Elevated MV gradient (>5 mmHg) | 3,346 (36.8) | 1,612 (30.8) | 1,737 (45.0) | <0.001 |
| Procedural success (MV gradient independent) | 8,095 (87.4) | 4,685 (88.0) | 3,410 (86.5) | 0.030 |
| Procedural success | 5,155 (55.7) | 3,260 (61.3) | 1,895 (48.1) | <0.001 |
| Values are mean ± SD or n (%)  MR: mitral regurgitation; MV: mitral valve | | | | |

# **Supplemental Table 5. Distribution of Sex and Race Before and After the COAPT Trial.**

| **Distribution by Sex** | **All Patients (N = 9,262)** | **Male (N = 5,321)** | **Female (N = 3,941)** | ***p* Value** |
| --- | --- | --- | --- | --- |
| Before COAPT (January 2013–August 2018) | 1,517 (16.4) | 879 (57.9) | 638 (42.1) | 0.690 |
| After COAPT (September 2018– December 2021) | 7,745 (83.6) | 4,442 (57.4) | 3,303 (42.6) |  |
| **Distribution by Race** | **All Patients (N = 9,232)** | **White (N = 7,424)** | **Black  (N = 1,504)** | ***p* Value** |
| Before COAPT (January 2013–August 2018) | 1,514 (16.4) | 1,270 (83.9) | 185 (12.2) | < 0.001 |
| After COAPT (September 2018– December 2021) | 7,723 (83.6) | 6,154 (79.7) | 1,319 (17.1) |  |
| Values are n (%) | | | | |

# **Supplemental Table 6. Outcomes Stratified by Functional Mitral Regurgitation Etiology (Ischemic vs. Non-Ischemic).**

| **Multivariable-adjusted Cox proportional hazard models.** | | | |
| --- | --- | --- | --- |
|  | **Adjusted Hazard Ratio 95% CI** | ***p* Value** | ***p* Value for Interaction*** |
| **Mortality** |  |  |  |
| Black (vs. White) | 0.92 (0.73-1.15) | 0.442 | 0.736 |
| Asian (vs. White) | 1.04 (0.72-1.51) | 0.828 |  |
| Female | 0.92 (0.77-1.09) | 0.327 | 0.519 |
| **Readmission** |  |  |  |
| Black (vs. White) | 1.07 (0.92-1.25) | 0.393 | 0.708 |
| Asian (vs. White) | 1.05 (0.77-1.43) | 0.768 |  |
| Female | 0.99 (0.85-1.14) | 0.864 | 0.483 |
| **Heart failure readmission** |  |  |  |
| Black (vs. White) | 1.35 (1.09-1.67) | 0.006 | 0.631 |
| Asian (vs. White) | 0.71 (0.41-1.23) | 0.222 |  |
| Female | 1.00 (0.81-1.25) | 0.981 | 0.243 |

*Interaction between ischemic MR etiology (defined as ischemic acute, post-infarction, or ischemic chronic) vs. non-ischemic/other etiologies (defined as non-ischemic dilated cardiomyopathy, restrictive cardiomyopathy, hypertrophic cardiomyopathy, or pure annular dilation).
